# The Value of Using Quantitative MRI based on Synthetic Acquisition and Apparent Diffusion Coefficient to Monitor Multiple Sclerosis Lesion Activity

**DOI:** 10.2174/0115734056343086250103020830

**Published:** 2025-01-09

**Authors:** Abdullah H. Abujamea, Fahad B. Albadr, Arwa M. Asiri

**Affiliations:** 1 Department of Radiology and Medical Imaging, King Saud University Medical City, King Saud University, Riyadh, Saudi Arabia

**Keywords:** Multiple scleroses, Quantitative MRI, Synthetic MRI, Gadolinium-based *contrast agents*, *Relaxation times*, *Proton density*, *Apparent diffusion coefficient*

## Abstract

**Background:**

Multiple sclerosis (MS) is one of the most common disabling central nervous system diseases affecting young adults. Magnetic resonance imaging (MRI) is an essential tool for diagnosing and following up multiple sclerosis. Over the years, many MRI techniques have been developed to improve the sensitivity of MS disease detection. In recent years synthetic MRI (sMRI) and quantitative MRI (qMRI) have gained traction in neuroimaging applications, providing more detailed information than traditional acquisition methods. These techniques enable the detection of microstructural changes in the brain with high sensitivity and robustness to inter-scanner and inter-observer variability. This study aims to evaluate the feasibility of using these techniques to avoid administering intravenous gadolinium-based contrast agents (GBCAs) for assessing MS disease activity and monitoring.

**Materials and Methods:**

Forty-two known MS patients, aged 20 to 45, were scanned as part of their routine follow-up. MAGnetic resonance image Compilation (MAGiC) sequence, an implementation of synthetic MRI, was added to our institute's routine MS protocol to automatically generate quantitative maps of T1, T2, and PD. T1, T2, PD, and apparent diffusion coefficient (ADC) data were collected from regions of interest (ROIs) representing normal-appearing white matter (NAWM), enhancing, and non-enhancing MS lesions. The extracted information was compared, and statistically analyzed, and the sensitivity and specificity were calculated.

**Results:**

The mean R1 (the reciprocal of T1) value of the non-enhancing MS lesions was 0.694 s^-1^ (T1=1440 ms), for enhancing lesions 1.015 s^-1^ (T1=985ms), and for NAWM 1.514 s^-1^ (T1=660ms). For R2 (the reciprocal of T2) values, the mean value was 6.816 s^-1^ (T2=146ms) for non-enhancing lesions, 8.944 s^−1^ (T2=112 ms) for enhancing lesions, and 1.916 s^−1^ (T2=522 ms) for NAWM. PD values averaged 93.069% for non-enhancing lesions, 82.260% for enhancing lesions, and 67.191% for NAWM. For ADC, the mean value for non-enhancing lesions was 1216.60×10^−6^ mm^2^/s, for enhancing lesions 1016.66×10^−6^ mm^2^/s, and for NAWM 770.51×10^−6^ mm^2^/s.

**Discussion:**

Our results indicate that enhancing and non-enhancing MS lesions significantly decrease R1 and R2 values. Non-enhancing lesions have significantly lower R1 and R2 values compared to enhancing lesions.

**Conclusion:**

Conversely, PD values are significantly higher in non-enhancing lesions than in enhancing lesions. For ADC, while NAWM has lower values, there was minimal difference between the mean ADC values of enhancing and non-enhancing lesions.

## 1 INTRODUCTION

*Multiple sclerosis (MS) is one of the most common disabling central nervous system diseases affecting young adults. Although it is more prevalent among young individuals, particularly women and those living in northern latitudes, MS can affect people of any age and gender. It is a chronic inflammatory and degenerative disease of the central nervous system, characterized by multifocal demyelination resulting from an autoimmune response to self-antigens in genetically susceptible individuals* [1].

Symptoms of MS can vary among patients and may include visual, motor, sensory, and cognitive impairments, leading to significant disability. The severity of the pathological process differs according to the stage and type of MS, including the location and progression of lesions.

Magnetic resonance imaging (MRI) is an essential tool for diagnosing and monitoring multiple sclerosis. Numerous MRI techniques have been developed to improve the sensitivity of disease detection, including T1, T2, T2*, diffusion imaging, spectroscopy, susceptibility-weighted imaging, and magnetization transfer imaging [2-5]. However, MR features are only moderately correlated with disability [6, 7]. Studies have reported poor to moderate correlations between disability and radiological features such as white matter lesion load [8, 9].

In multiple sclerosis, many parameters have been investigated as potential quantitative measurements. One parameter that has been intensively studied in recent years is myelin water content [10-12]. Other studies have demonstrated relationships between various MR parameters, such as relaxation times, total water content, magnetization exchange properties among different proton pools, and disease activity [11, 13, 14]. Several studies have reported differences in quantitative MR parameters between MS subjects and healthy controls [4, 15].

In addition to diffuse white matter changes and atrophy, which are predictors of disability and short-term disease progression in MS [16], contrast-enhanced MRI is a well-established method for assessing disease status and activity [17]. Contrast-enhanced lesions indicate a breakdown of the blood-brain barrier and an inflammatory response, known to occur in acutely demyelinating lesions [18, 19].

The safety of gadolinium-based contrast agents (GBCAs) used in MRI has become a major concern in radiology in recent years. In addition to the risk of developing nephrogenic systemic fibrosis (NSF) in patients with acute kidney injury or stage 4 or 5 chronic kidney disease (CKD) [20], recent studies have provided evidence of gadolinium deposition in the brain following multiple contrast-enhanced brain MRIs [21-24]. Patients with MS are likely to undergo repeated contrast-enhanced MR imaging throughout their lives to monitor disease status and activity; therefore, they are at a higher risk for cumulative gadolinium deposition due to the relatively early onset of the disease. Furthermore, the economic costs associated with GBCA, the staff required to administer it, the increased scanning time, and the discomfort experienced by patients due to intravenous injections all contribute to the need to minimize the amount of GBCA that MS patients receive.

In recent years, synthetic MRI has gained traction in various neuroimaging applications [25, 26]. Compared to traditional MRI, synthetic MRI relies on the simultaneous acquisition of T1, T2, and proton density (PD) images, resulting in shorter scan times and more comprehensive information than traditional acquisition methods. Additionally, quantitative data can be considered independent of the scanner type or imaging protocol used [27]. The notable benefit of quantitative MR imaging (qMRI) is its ability to generate spatial maps where each voxel corresponds to a quantitative value that reflects the physical properties of the studied tissues, such as free water proportion, myelination, or iron content. These properties are represented in quantitative maps such as R1 (the reciprocal of T1), R2 (the reciprocal of T2), R2* (the reciprocal of T2*), and quantitative susceptibility mapping (QSM) [28, 29].

While conventional MR imaging plays an important role in diagnosing and monitoring therapy for MS, it provides limited information regarding the pathophysiology of tissue damage because traditional sequences are unable to detect subtle alterations in white matter (WM) and gray matter (GM). Quantitative MR imaging (qMRI) addresses these gaps, detecting microstructural changes in the brain with great sensitivity and robustness to inter-scanner and inter-observer variability.

Moreover, due to its independence from external influences, qMRI may provide insights that improve the diagnostic accuracy of multiple sclerosis and its underlying pathology. qMRI can also serve as a stable and consistent follow-up tool for monitoring disease progression, as regular MR scans may be required over decades.

Diffusion-weighted imaging (DWI) which shows the mobility of water molecules in tissues can serve as a marker of MS disease activity and progression [4]. Myelin breakdown may be predicted using apparent diffusion coefficient (ADC) maps, a quantitative measurement derived from DWI [30]. During both active and chronic phases of MS, the mean ADC values of lesions may change depending on the type of edema present [31].

Quantification in modern diagnostic imaging is becoming increasingly essential, especially in the era of artificial intelligence (AI), where precise, measurable data can significantly enhance disease prediction, diagnosis, and personalized treatment. Quantitative imaging transforms medical scans into rich datasets with specific metrics, such as tissue volume, density, texture, and functional parameters. These data points enable clinicians and AI algorithms to identify subtle changes that might be overlooked through traditional qualitative assessments, facilitating earlier and more accurate detection of diseases like cancer, cardiovascular disease, and neurodegenerative disorders. [32-34]

The purpose of this study is to utilize recent MRI techniques based on synthetic MRI to investigate the visibility of extracted MRI parameters, such as relaxation properties and proton density, and to combine these with DWI to eliminate the need for intravenous contrast agents in assessing MS disease activity. This approach may also benefit patients with poor renal function or those allergic to gadolinium chelates, as it avoids the usage of gadolinium-based contrast agents.

## 2 MATERIALS AND METHODS

This study was conducted under the principles of the Declaration of Helsinki. Approval was granted by the Ethics Committee of King Saud University Medical City’s institutional review board (Ref. No. 16/0427/IRB). Forty-two patients with multiple sclerosis (MS) fulfilling the McDonald diagnostic criteria [35] (16 males and 26 females) aged 20 to 45 years (median age = 29) were scanned as part of their routine follow-up MRI scanning protocol at the Department of Radiology at King Khalid University Hospital in Riyadh, Saudi Arabia. Table **1** provides details of patient demographics.

A GE Optima MR 450w GEM 1.5 T (GE Healthcare, Milwaukee, WI) scanner was used with a standard Head/Neck 20 Channel RF Coil. The MAGnetic resonance image Compilation (MAGiC) sequence, which is an implementation of syntactic MRI image processing, was added to our institution's routine MS protocol to automatically generate quantitative maps of T1, T2, and PD. Our MS protocol includes axial T2, diffusion-weighted (DW), sagittal 3D T1 pre- and post-contrast, and axial T1 post-contrast sequences. The MAGiC sequence was acquired in the axial plane pre-contrast with the following parameters: a field of view of 240 × 240 mm^2^, a slice thickness/gap of 4.5/0.5 mm, an image matrix of 320 × 224, TE/TR of 4500/26 ms, TI of 12.3 ms, and an echo train length of 12. For axial DW, the parameters were similar to those used in the MAGiC sequence, except for TE/TR of 5500/77, an imaging matrix of 128 × 128, and b-values of 0/1000. For the contrast-enhanced axial T1 SE sequence, the parameters were also similar to those of the MAGiC sequence, except for TE/TR of 400/10 and a matrix of 320 × 192. After completing the acquisition of pre-contrast sequences, the MAGiC sequence was acquired with an approximate time of six minutes. Patients were then instructed not to move their heads, and a Dotarem® (gadoterate meglumine, Guerbet, Roissy CdG, France) contrast agent was injected intravenously as a bolus (0.2 mL/kg of body weight). T1 post-contrast images were acquired at least 10 minutes after contrast injection.

### 2.1 MR Image Post-processing

The scanner was configured to automatically generate T1, T2, PD, R1 (=1/T1), and R2 (=1/T2) quantitative maps upon completion of the MAGiC sequence acquisition. The maps were then transferred to the AW VolumeShare 7 (GE Healthcare) diagnostic station, where intrinsic parameters such as R1, R2, and PD values were correlated with the lesion’s ADC value. Parameters were also calculated from the normal-appearing white matter (NAWM) in the contralateral hemisphere. This approach was taken to avoid discrepancies caused by the locations of lesions within the brain's white matter, which could affect the accuracy of the parameters, as well as to mitigate the impact of patient aging on the quantitative MRI parameters [35].

A circular region of interest (ROI) was manually placed in the center of the MS lesion, including the enhancing portion if it was visible post-contrast. The ROI was positioned slightly within the visual outer rim of the lesion to prevent partial volume effects from surrounding tissue. The ROI size varied based on the lesion size, with the average ROI size ranging from 5 to 20 mm^2^. Fig. (**1**) illustrates the placement of the ROIs on selected images of MS patients.

MS lesions were collected from various locations in the brain, including the periventricular white matter, juxtacortical region, and infratentorial region. Images were shown side by side to experienced neuroradiologists for each patient, and MS lesions were identified as enhancing or non-enhancing through visual assessment. All enhancing lesions were recorded, and information was extracted from the corresponding images. Ring-like or faintly enhancing lesions were excluded from the total count to avoid controversy or suspicion. For non-enhancing lesions, lesions from different brain locations were selected, and information was extracted from the corresponding images. Using rigid registration in the AW workstation, diffusion and post-contrast images were registered to synthetic fluid-attenuated inversion recovery (FLAIR), T2, phase-sensitive, and parametric map images generated by the MAGiC sequence. This registration allowed for the accurate mapping of ROIs in the post-contrast images to their corresponding values in the other images, as shown in Fig. (**2a** and **b**).

### 2.2 Data Analysis and Statistics

Following the calculation of mean values for intrinsic parameters and ADC values for each ROI, the resulting means were compared using a mixed linear model. Quantitative measurements of enhancing lesions and non-enhancing lesions *versus* normal-appearing white matter (NAWM) were statistically analyzed, and the sensitivity and specificity were calculated. Receiver operating characteristic (ROC) curve analysis based on logistic regression was performed on the data to assess the optimal sensitivity and specificity cutoff values for the four parameters: T1, T2, PD, and ADC. The corresponding area under the ROC curve (AUC) was reported. All statistical analyses were conducted using Microsoft Excel 2019.

## 3 RESULTS

A total of two hundred and thirty MS lesions were detected, of which only seventy-two were enhancing post-contrast. Lesions with faint enhancement or those showing only ring enhancement were not included in this count. Data from the corresponding normal-appearing white matter (NAWM) in the contralateral hemisphere were collected from the patient's MRI images. These lesions were obtained from forty-two individuals. Seventy-two regions of interest (ROIs) were drawn in enhancing MS lesions, and one hundred fifty-eight ROIs were drawn in non-enhancing MS lesions. An additional two hundred and thirty ROIs were drawn in the corresponding NAWM in the contralateral hemisphere, matching the location and size of the corresponding MS lesions. Fig. (**1**) shows the placement of the ROIs on the selected images of MS patients.

The results shown in Table **2** demonstrate that the mean R1 value of non-enhancing MS lesions (0.694 s^−1^, T1 = 1440 ms) is significantly lower than that of enhancing lesions (1.015 s^−1^, T1 = 985 ms) and NAWM tissue (1.514 s^−1^, T1 = 660 ms). Regarding R2 values, non-enhancing lesions have lower mean R2 values (6.816 s^−1^, T2 = 146 ms) compared to enhancing MS lesions (8.944 s^−1^, T2 = 112 ms) and NAWM (1.916 s^−1^, T2 = 522 ms). For PD, non-enhancing MS lesions have higher mean PD values (93.069%) compared to enhancing MS lesions (82.260%) and NAWM (67.191%). As far as ADC is concerned, the mean values of non-enhancing MS lesions have higher ADC values (1216.60 × 10^−6^ mm^2^/s) compared to enhancing MS lesions (1016.66 × 10^−6^ mm^2^/s) and NAWM (770.51 × 10^−6^ mm^2^/s).

When comparing the mean ratios of enhancing and non-enhancing lesions to the NAWM in the contralateral hemisphere, the mean R1 and R2 ratios show higher values for enhancing MS lesions compared with non-enhancing MS lesions. For R1, the ratio was 0.640 for enhancing lesions compared to 0.475 for non-enhancing lesions. For R2, the mean ratio for enhancing MS lesions was 0.745 compared to 0.577 for non-enhancing MS lesions. For PD, the mean ratio shows higher values for non-enhancing MS lesions (1.373) compared to 1.267 for enhancing MS lesions. Regarding ADC, the mean ratio shows higher values for non-enhancing MS lesions (1.561) compared to 1.367 for enhancing MS lesions.

Receiver operating characteristic (ROC) curve analysis based on logistic regression was performed on the data to assess the optimal sensitivity and specificity cutoff values for the four parameters: T1, T2, PD, and ADC, as shown in Fig. (**3**). The distributions of mean R1, mean R2, mean PD and mean ADC for enhancing and non-enhancing lesions are shown in Fig. (**4**). As can be seen from Fig. (**4**), most enhancing MS lesions have R1 values between 0.9 s^−1^ and 1.2 s^−1^; below 0.8 s^−1^, it is rare to observe an enhancing lesion. For R2, most enhancing lesions show higher values, exceeding 8.5 s^−1^; it is unlikely to find enhancing lesions with R2 values below 7.5 s^−1^. For PD, most enhancing lesions exhibit low PD values ranging from 75% to 90%, while non-enhancing lesions tend to have higher PD values exceeding 90%. On the other hand, ADC generally shows lower values for enhancing lesions (900 - 1200 × 10^−6^ mm^2^/s) compared to non-enhancing lesions, where ADC values tend to be higher (more than 1300 × 10^−6^ mm^2^/s). Table **2** presents the sensitivity and specificity for a set of f threshold cutoff values for R1, R2, PD, and ADC for enhancing, non-enhancing, and NAWM tissues. For R1, the best cutoff value was 0.82 s^−1^, with sensitivity and specificity of 90.28% and 94.94%, respectively. For R2, the best cutoff value was 8 s^−1^, with sensitivity and specificity of 84.72% and 91.77%, respectively. For PD, the best cutoff value was 88%, with sensitivity and specificity of 83.33% and 82.28%, respectively. For ADC, the best cutoff value was 1120 × 10^−6^ mm^2^/s, with sensitivity and specificity of 80.56% and 64.55%, respectively (Table **3**).

## 4 DISCUSSION

Quantitative MRI is poised to play a transformative role in the future of diagnostic imaging, offering precise, reproducible measurements that extend beyond the qualitative assessments typical of conventional MRI. As a result, quantitative MRI holds great promise for improving diagnostic accuracy, monitoring disease progression, and personalizing treatment for various neurological and systemic conditions. In diseases such as multiple sclerosis (MS), Alzheimer’s disease, [34] and certain cancers, quantitative MRI can reveal early pathological changes even before clinical symptoms manifest, allowing for earlier intervention. [36]

Due to the pathological heterogeneity of MS lesions, conventional MR methods without the aid of MR contrast agents are not specific to the diagnostic workup of MS patients. Thus, alternative MR methods such as quantitative PD, T1, and T2 relaxation time analysis [37], magnetization transfer ratio [38], apparent diffusion coefficient (ADC) [39], diffusion tensor imaging [40], quantitative susceptibility mapping (QSM) [29] and MR spectroscopy has been proposed to characterize lesions in MS patients [41].

In this study, we aimed to utilize quantitative MRI to evaluate the visibility of using tissue intrinsic parameters such as T1, T2, PD, and ADC values to differentiate between active and inactive MS lesions without the need for intravenous MRI contrast agents. In addition to standard MRI image weighting techniques such as T1, T2, PD, FLAIR, and double inversion recovery (DIR), synthetic MRI provides quantitative maps of tissue parameters like R1, R2, and PD, which we used to quantify normal and diseased tissues.

Following the disruption of the blood-brain barrier and immune cell infiltration in early active lesions, inflammatory activity blooms from venules, leading to progressive demyelination and axonal loss with a centrifugal spread. These processes are paralleled by the pattern of enhancement observed after gadolinium delivery on MR imaging [42, 43]. As inflammation progresses due to myelin breakdown and edema, cellular infiltrates increase, resulting in decreased R1 and R2 values, and increased PD values within lesions compared to normal-appearing white matter (NAWM) [44-46].

Our results show that while both enhancing and non-enhancing MS lesions exhibit a significant decrease in R1 and R2 values, non-enhancing MS lesions have significantly lower R1 and R2 values compared with enhancing MS lesions. Conversely, for PD, while both enhancing and non-enhancing MS lesions show significant increases in PD values, non-enhancing MS lesions have significantly higher PD values compared to enhancing MS lesions. This finding is consistent with the results of other studies [47-49]. Tranfa *et al*. described the progression stages of MS lesions and the pattern of enhancement from early active lesions, characterized by a typical nodular enhancement pattern, through late active lesions with a peripheral pattern of enhancement, to eventually a chronic inactive stage characterized by the absence of gadolinium enhancement [43].

For ADC, the situation is more complex and depends on the age of the MS lesions, which we did not account for in our study [47, 48]. Water diffusion changes in MS are observed as a consequence of tissue damage, and diffusion-weighted imaging (DWI) can therefore be used to quantitatively assess tissue damage [30]. Before blood-brain barrier leakage and MS lesions appear, the DWI and ADC maps may show alterations. In MS MRI scans, DWI is added to the standard MRI sequences to assess the severity of MS and its extension into NAWM [49]. A restricted ADC is noted in the early stages of acute lesions, but these values subsequently normalize or increase. Restriction is believed to develop before the lesions' contrast enhancement becomes observable and normalizes long before enhancement disappears [50]. Microscopic injuries are also seen in NAWM, not only in visible lesions [49, 50]. According to Werring *et al*., ADC values of pre-lesion NAWM rise approximately six months before contrast-enhancing lesions appear, while ADC values of contralateral NAWM remain unchanged [51]. Our results show plausible agreement with previous studies that reported a significant increase in ADC values of chronic lesions compared with NAWM [30, 31]. Unlike the findings of Terzi *et al*., which reported no significant difference between ADC values of active and chronic lesions [11], our study showed that non-enhancing MS lesions exhibit significantly increased ADC values compared to enhancing lesions. This may be partially explained by the fact that we did not consider the age of the MS lesions, as other studies indicate that lesion age can affect ADC values. It was observed that old lesions not showing enhancement post-contrast administration had very high ADC values (greater than 1300 × 10^−6^ mm^2^/s), which may explain the overall high ADC values of non-enhancing MS lesions.

## CONCLUSION

In summary, this study demonstrates the potential of quantitative MRI metrics, including T1, T2, PD, and ADC values, as adjunct tools to conventional contrast-enhanced imaging for differentiating between active and inactive MS lesions. Our findings indicate that non-enhancing MS lesions exhibit significantly lower R1 and R2 values and higher PD values compared with enhancing lesions, reflecting changes associated with chronic tissue damage. This aligns with the observed progression of MS lesions, where inflammatory activity and tissue changes lead to variations in MRI relaxation times. Additionally, while ADC values showed an increase in chronic lesions, the differentiation between active and inactive lesions may be complicated by lesion age, as ADC values fluctuate with the lesion’s stage of progression.

The ability to characterize MS lesions with quantitative metrics without relying solely on gadolinium-based contrast agents holds promise for improving the safety and accessibility of MS diagnostic imaging. By quantifying intrinsic tissue parameters through synthetic MRI, clinicians can gain additional information about lesion pathology and disease progression while reducing the need for contrast administration. Further research should explore the potential for quantitative MRI metrics to track lesion age and assess subclinical changes in normal-appearing white matter. Ultimately, these advancements could enhance our understanding of MS pathology, refine diagnostic protocols, and provide new avenues for monitoring disease activity and therapeutic response in MS patients.

There are several limitations to this study. First, since our sample size is smaller, this may limit the statistical power and generalizability of our conclusions.. In the current study, we aimed to provide preliminary insights into our hypothesis, and we believe that expanding the sample size in subsequent work will strengthen the validity of our conclusions. Second, we did not consider the activity and age of the MS lesions or when the patient started experiencing an acute MS attack. We recognize that these variables can influence quantitative MRI parameters. While our current study focused on lesions post-contrast enhancement, we believe incorporating these variables in future analyses could provide a deeper understanding of how they affect R1, R2, PD, and ADC values, also we believe that the exclusion of certain ring-enhancing and faintly enhancing lesions may impact the robustness of our results so a future study could benefit from quantifying enhancement characteristics. Third, the limitation of using only a single field strength may restrict the generalizability of our findings across different MRI field strengths. Future studies using multiple field strengths could provide a more comprehensive evaluation of the robustness of our findings and ensure broader applicability across different MRI systems. Finally, the absence of histopathological validation may limit our ability to confirm the imaging findings at a cellular or molecular level, which is critical for a comprehensive understanding of lesion pathology.

## Figures and Tables

**Fig. (1):**
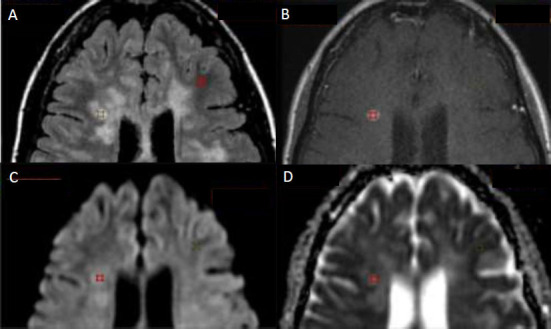
The locations of the region of interest (ROI) within the MS lesions, (**A**) Axial T2 fluid-attenuated inversion recovery (FLAIR) image, (**B**) axial SE T1 post-contrast image, (**C**) DWI image and (**D**) ADC image. The ROIs are located in the enhancing lesion and in the NAWM tissue.

**Fig. (2a):**
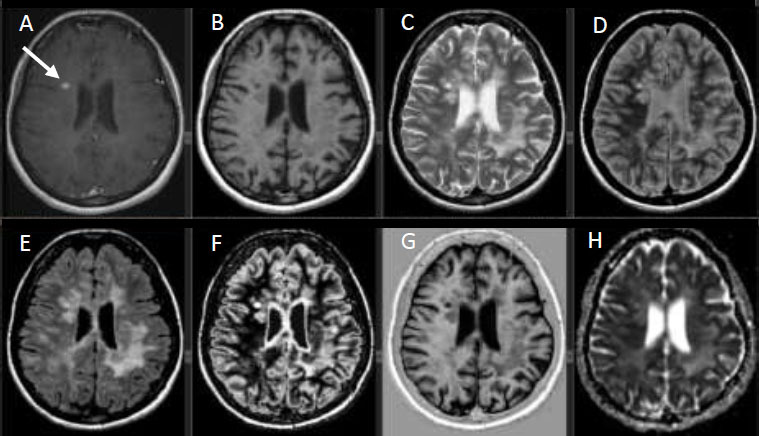
The registration of T1 post-contrast images with ADC and synthetic MRI scan, (**A**) Axial T1 post-contrast image, (**B**) Axial T1 pre-contrast image, (**C**) Synthetic Axial T2 image, (**D**) Synthetic PD image, (**E**) Synthetic FLAIR image, (**F**) Synthetic double inversion recovery (DIR) image, (**G**) Synthetic phase-sensitive image and (**H**) ADC image. The white arrow points to the enhancing MS lesion.

**Fig. (2b):**
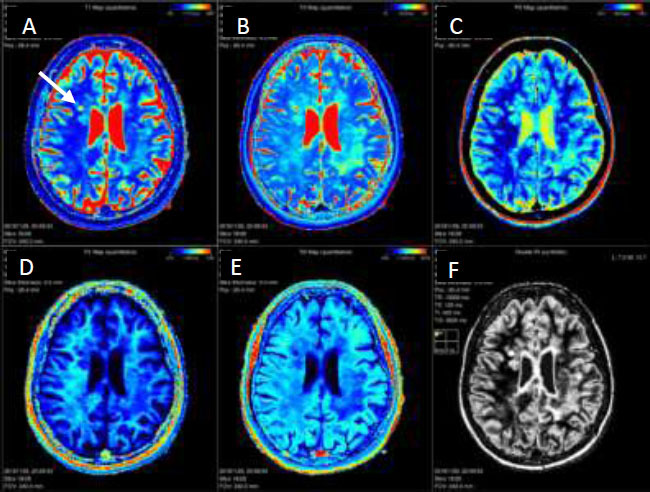
The registration of the images in Fig. (**2a**) with the corresponding quantitative MRI maps, (**A**) Axial T1 map, (**B**) Axial T2 map, (**C**) Axial PD map, (**D**) Axial R1 map, (**E**) Axial R2 map and (**F**) Synthetic double inversion recovery (DIR) image. The white arrow points to the enhancing MS lesion.

**Fig. (3):**
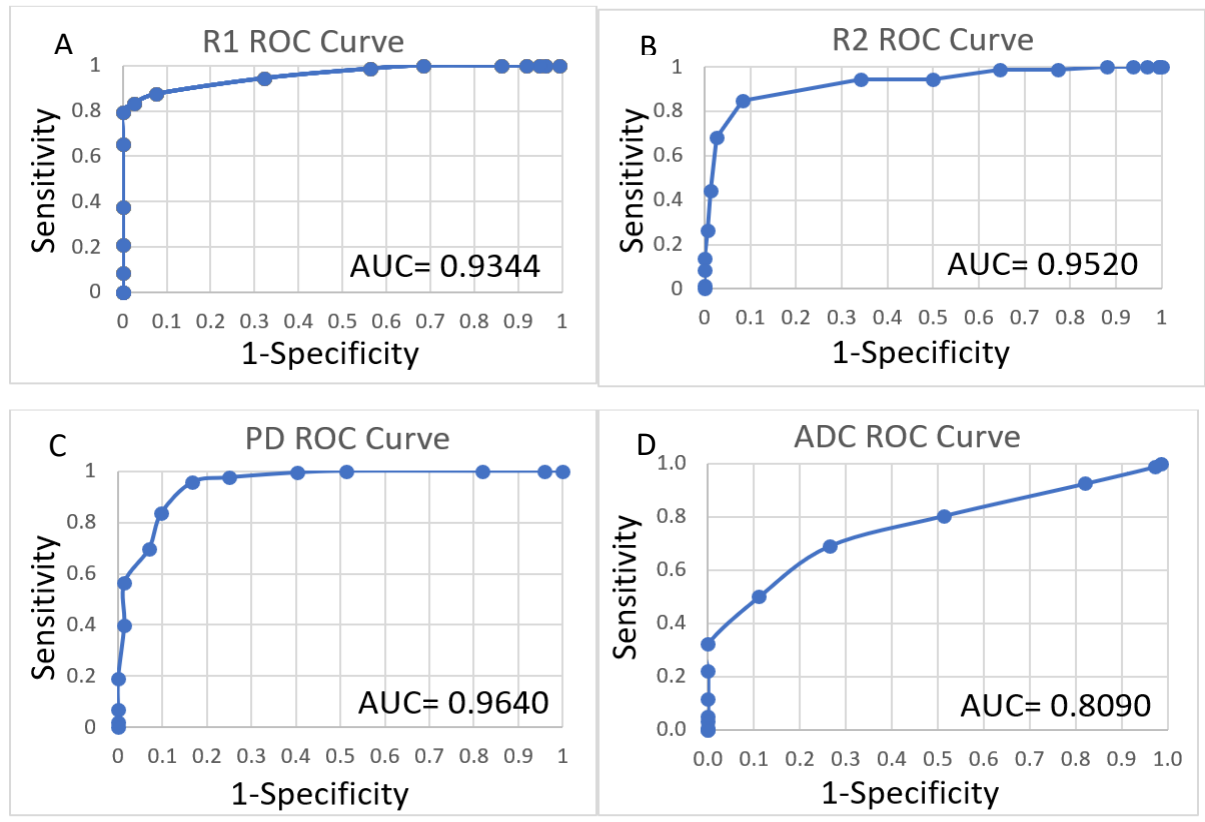
Receiver operating characteristic (ROC) curve analysis of R1 (**A**), R2 (**B**), PD (**C**) and ADC (**D**) indicating area under the curve (AUC).

**Fig. (4):**
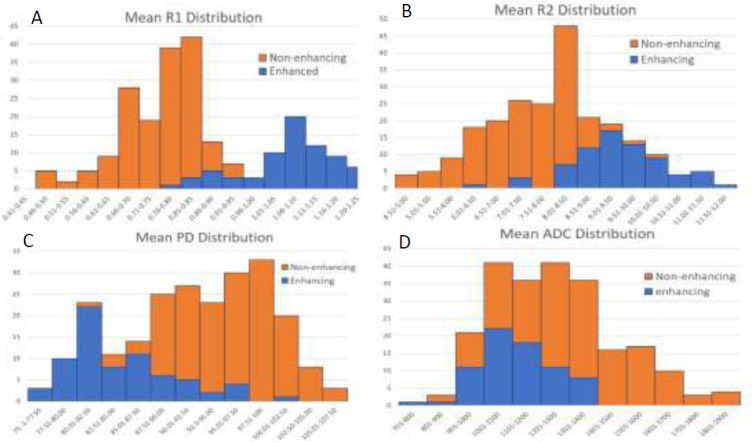
**A**, Histograms of R1 distribution for enhancing and non-enhancing lesions. **B**, Histograms of R2 distribution for enhancing and non-enhancing lesions. **C**, Histograms of PD distribution for enhancing and non-enhancing lesions. **D**, Histograms of ADC distribution for enhancing and non-enhancing lesions.

**Table 1:** Patient demographics.

Number of patients	42
Age (Years)	29.3 ± 6.4 (Range, 20-45)
Sex (M/F)	16/26
Disease duration (years)	5.6 ± 4.6

**Note: **Data are shown as mean ± SD unless otherwise specified.

**Table 2:** Quantitative measurements of normal-appearing white matter, enhancing and non-enhancing MS lesions before and after gadolinium-based contrast agent injection.

-	R1 (s^-1^) ± SD	R2 (s^-1^) ± SD	PD (au) ± SD	ADC (10^-6^ mm^2^/s) ± SD
Non-enhancing MS lesions (n=159)	0.694 ± 0.10	6.816 ± 1.18	93.069 ± 4.87	1216.60 ± 232
Enhancing MS lesions (*n*=72)	1.015 ± 0.11	8.944 ± 1.09	82.260 ± 5.54	1016.66 ± 127
NAWM (*n*=230)	1.514 ± 0.17	11.916 ± 1.07	67.191 ± 5.12	770.51 ± 72.10
Enhancing MS lesions/ NAWM	0.640 ± 0.07	0.745 ± 0.10	1.267 ± 0.10	1.367 ± 0.21
Non-enhancing MS lesions/ NAWM	0.475 ± 0.10	0.577 ± 0.10	1.373 ± 0.13	1.561 ± 0.30

**Note: **Data are means ± SD. P < .001 for each metric among enhancing, non-enhancing, and NAWM tissues.

**Table 3:** Sensitivity and specificity for different cutoffs for R1, R2, PD, and ADC values to predict enhancing MS lesions.

**Cut off Values**	**Sensitivity (%)**	**Specificity (%)**	**PPV (%)**	**NPV (%)**	**Accuracy (%)**
**R1 (**s^-1^)					
0.78	98.61	82.28	71.71	99.24	87.39
0.80	94.44	92.41	85	97.33	93.04
0.82	90.28	94.94	89.04	95.54	93.47
**R2 (**s^-1^)					
7.8	93.06	81.66	69.79	96.26	85.31
7.9	90.28	89.24	79.26	95.27	89.56
8	84.72	91.77	82.43	92.94	89.56
**PD (%)**					
87	80.56	87.97	75.32	90.85	85.65
88	83.33	82.28	68.18	91.55	82.60
89	86.11	75.95	62	92.31	79.13
**ADC (**10^-6^ mm2/s)					
1100	73.61	68.99	51.96	85.16	70.43
1120	80.56	64.55	50.87	87.93	69.56
1140	81.94	60.75	48.76	88.07	67.39

**Abbreviations: **PPV, positive predictive value; NPV, negative predictive value.

## Data Availability

The data and supportive information are available within the article.

## LIST OF ABBREVIATIONS

MS: Multiple Sclerosis
MRI: Magnetic Resonance Imaging
NSF: Nephrogenic Systemic Fibrosis
PD: Proton Density
qMRI: quantitative MR imaging
QSM: Quantitative Susceptibility Mapping
WM: White Matter
GM: Gray Matter
DWI: Diffusion-weighted Imaging
AI: Artificial Intelligence
ADC: Apparent Diffusion Coefficient
MAGiC: MAGnetic resonance image Compilation
NAWM: Normal-appearing White Matter
ROI: Region of Interest
DIR: Double Inversion Recovery
FLAIR: Fluid-attenuated Inversion Recovery
ROC: Receiver Operating Characteristic

## STANDARDS OF REPORTING

STROBE guidelines were followed.
